# Parliament and the Profession

**Published:** 1917-04-14

**Authors:** 


### Parliament and the Profession.

### Sickness in Military Camps.

MR. MACPHERSON informed Mr. Haydn Jones that there had been an outbreak of sickness in certain camps. At Kinmel Park the prevalent illness was influenzal in character. There is hospital accommodation for 600 patients, and there have been thirteen deaths. At Litherland during February and the first half of March the average hospital admissions were 5.5 per day, and there were seven deaths. At Park Hall influenzal pneumonia was prevalent. Medical inspection of the camps produced a satisfactory report as to sanitation.

### National Insurance Advisory Committee.

SIR EDWIN CORNWALL, representing the National Health Insurance Commissioners, stated that the Advisory Committee to the National Health Insurance Joint Committee had been reduced from a total membership of 168 to about thirty, in order to save expense and obtain a more workmanlike body. The Commissioners are prepared to add one or two more doctors to the Committee, or, alternatively, to appoint a small separate Medical Advisory Committee, and representatives of the medical profession are being consulted on the subject.

### Distribution of Appeals for Charities.

MR. RAWLINSON asked the President of the Board of Trade whether the Order against the distribution of advertising circulars is intended to apply to circulars appealing for funds for charitable purposes or whether it is confined to circulars offering goods for sale; he was informed that a general licence has been issued by the Royal Commission on Paper permitting for the present the dispatch and delivery of circulars other than those of traders.

